# Molecular Approach for Tracing Dissemination Routes of Shiga Toxin-Producing *Escherichia coli* O157 in Bovine Offal at Slaughter

**DOI:** 10.1155/2014/739139

**Published:** 2014-01-30

**Authors:** Hiroshi Asakura, Kazuya Masuda, Shigeki Yamamoto, Shizunobu Igimi

**Affiliations:** ^1^Division of Biomedical Food Research, National Institute of Health Sciences, Kamiyoga 1-18-1, Setagaya-ku, Tokyo 158-8501, Japan; ^2^Department of Fisheries, Tokai University, 3-20-1 Orido, Shimizu-ku, Shimizu-shi, Shizuoka 424-8610, Japan

## Abstract

Bovine offal is currently recognized as one of the sources of human Shiga toxin-producing *Escherichia coli* (STEC) infection in Japan. Here, the prevalence and genetic characterization of STEC O157 in bovine feces, offal, and carcasses at slaughtering were examined between July and October in 2006. STEC O157 was detected in 31 of 301 cattle feces (10.3%) delivered from 120 farms. Simultaneously, 60 bovine-originated offal (tongue, liver, and omasum) and carcasses were randomly selected and the detection of O157 STEC was examined as well. STEC O157 was isolated from 4 tongues (6.7%), 1 liver (1.7%), 3 omasa (5.0%), and 2 carcasses (3.3%), respectively. All the O157 isolates were positive for *eae* and *hlyA* genes, and 37 of 41 isolates (90.2%) exhibited *stx2c* genotype. PFGE analysis revealed the identical macrogenotypes of 4-tongue- and 1-liver-originated isolates and among 2 fecal isolates from animals slaughtered consecutively. Considering their continuous detection according to the slaughtering order, we concluded that these distributions of O157 in bovine offal and feces might be due to cross-contamination at (pre)slaughter. Our data thus reposes implication of better sanitary control in diapedesis from both upper and lower sites to prevent spread of this pathogen to bovine offal at slaughtering.

## 1. Introduction

Shiga toxin (Stx)-producing *Escherichia coli *(STEC) is one of the major foodborne pathogens that causes diarrheal illness in humans worldwide. Among a number of serotypes categorized in the STEC, O157 is the most major serotype associated with human infection [1]. Infection with STEC O157 can be achieved at least in part through the intake of contaminated foods, in which dairy products and meats contaminated with animal feces or intestinal contents during/after slaughtering are considered as the most common sources [2].

In Japan, bovine offal which include liver, heart, tongue and intestines are customarily eaten, a part of which are consumed raw and the contamination with pathogenic microorganisms in these meat products is therefore considered a high risk for human health. Indeed, as epidemiological records for foodborne O157 infections in Japan, 6 of 52 cases (11.5%) were associated with the bovine offal in years 2010-2011 [3]. More recently, we examined the prevalence of STEC in retail bovine offal products in Japan, revealing that 38 of 229 samples (16.6%) were positive for *stx* gene and four O157 and one O26 STEC were finally isolated from small intestine and omasum products [4]. However, the routes of contamination in these products remain unclear, especially the issue of how and whether cross-contamination might occur during slaughtering processes.

Given the background, here we examined the prevalence and genetic characterization of STEC O157 in bovine feces and offal at slaughterhouse in Japan.

## 2. Materials and Methods

### 2.1. Sampling, Isolation, and Identification of STEC O157

Fecal samples were collected with cotton swab from a total of 301 bovine from 120 farms between July and September, 2006, at a slaughterhouse in Japan. Simultaneously, 60 of these animals were randomly selected and 100 cm^2^ surface areas of their offal (livers, tongues, and omasum) and carcasses were swabbed, thereby being subjected to the detection procedure of STEC O157 as well. The swab samples were incubated in 10 mL of novobiocin-supplemented mEC broth (Eiken Kagaku, Tokyo, Japan) at 42°C for 24 h. The cultures were then subjected to screening of O157 using Path-Stik *E. coli* O157 (Celsis, Cambridge, UK) and mini-VIDAS (bioMérieux-Vitek, France). The O157-positive culture samples were then plated on CT-SMAC (Eiken Kagaku, Tokyo, Japan), CHROMagar O157 (CHROMagar, Paris, France), and Rainbow agar O157 (Biolog, Hayward, CA, USA). After incubation at 37°C for 24 h, suspected colonies were biochemically and/or genetically identified to be STEC O157 with API-20 kit (bioMérieux), O157 PCR screening set (Takara Bio, Shiga, Japan), and NH immunochromatography (Nippon-Ham, Tokyo, Japan) accordingly. The above culturing flow for each sample was started immediately within the day of slaughter.

### 2.2. Genetic Characterization and Toxin Production of STEC O157 Isolates

To characterize virulence gene possession of these isolates, total DNA was extracted from bacterial isolates with DNeasy kit (Qiagen, Hilden, Germany) according to the manufacturer's instruction. The *eaeA *and *hlyA *(*ehxA*) genes were detected by PCR using primers as described previously [5, 6]. The *stx* genes were subtyped by PCR as described [4, 7]. Shiga toxin (Stx) production was assayed by VTEC-RPLA (Denka Seiken, Tokyo, Japan).

### 2.3. Pulsed-Field Gel Electrophoresis (PFGE)

Representative O157 isolates were subjected to PFGE with *Xba*I endonuclease (New England BioLabs, Ipswich, MA, USA) using the CHEF Mapper system (Bio-Rad Laboratories, Hercules, CA, USA) as described previously [8]. The gel images were obtained using ethidium bromide stain. The electrophoretic patterns from PFGE were compared based on band position using FingerPrinting II software (Bio-Rad Laboratories, Hercules, CA, USA) and derived using the Dice coefficient with a maximum position tolerance of 1%. The strains were clustered using the unweighted pair group (UPGMA) method with arithmetic averages according to the manufacturer's instructions.

## 3. Results and Discussion

### 3.1. Dynamics for the Prevalence of STEC O157 at Slaughter

The majority of primary STEC infections are considered to be food- or water-borne, in which bovine and its products are one of the main sources of infection [9]. Epidemiological studies have mount evidence for the high prevalence of STEC especially O157 serotype in cattle intestines [10–12]. Throughout the screening tests herein, STEC O157 was detected from 31 of 301 samples from bovines feces that were slaughtered between July and September in 2006 in Japan (Table 1), of which 6 isolates (isolates # 7–12) were originated from animal slaughtered at the same day, even though they were delivered from different farms (Table 1). 60 offal (tongues, livers, and omasum) and carcass samples were simultaneously subjected to the O157 detection tests, resulting in that the STEC O157 was isolated from 4 tongues (6.7%), 1 liver (1.7%), 3 omasa (5.0%), and 2 carcasses (Table 1). Among them, 4 tongues and 1 liver isolates (isolates number 1–5) were originated from samples consecutively slaughtered (Table 1). Thus, these data showed the prevalence of STEC O157 in bovine feces and offal at slaughter. The consecutive detection of O157 from fecal and offal samples (i.e., isolates #1–3, 5) suggested that these isolates might be originated from identical sources.

The prevalence date herein at 10.5% is likely to be similar to that in a previous nationwide study in Japan [13]. Comparatively, little is known about the prevalence of STEC O157 in bovine offal at slaughter worldwide. Such biased information is likely to depend on the intake custom, as the individuals who eat them raw are limited and very few in westerns. Our data herein thus provided the implication of these foods for microbial risks to human infection.

### 3.2. Genetic Characterization of STEC O157 Isolates

Genetic characterization assays showed that all isolates were positive for *eaeA* and *hlyA* (Table 1). The most frequent *stx* subtype was *stx2c* (37 of 41, 90.2%), followed by *stx1*+*stx2c* (isolates number 6 and #21, 4.9%), and *stx1* (isolate #12, 2.4%), *stx1*+*stx2* (isolate #41, 2.4%), respectively (Table 1). The high yields of *stx2c* among the bovine isolates herein are in agreement with the previous reports [13]. We confirmed the Stx production in all isolates by VTEC-RPLA. Thus, these data indicated the potent virulence of these isolates.

### 3.3. Macrogenotypes of Representative O157 Isolates

**Figure 1:**
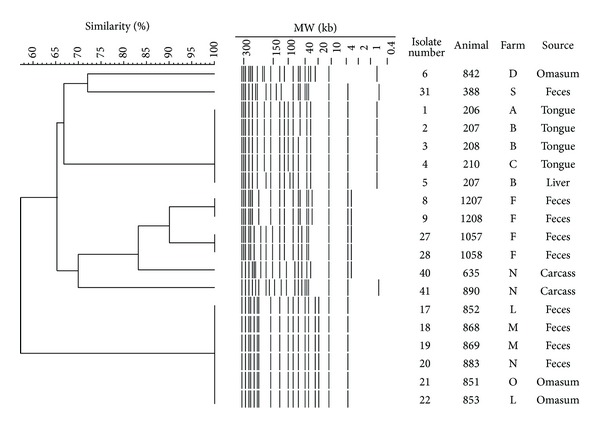
Pulsed-field gel electrophoresis (PFGE) patterns of representative STEC O157 isolates from bovine samples at slaughter. UPGMA dendrogram was constructed with the use of FingerPrinting II software.

Having nonconsecutive isolation of O157 STEC from fecal samples originated from identical farm (i.e., isolates #8, 9, 27, and 28 from farm F) and almost consecutive detection from tongue/liver (i.e., isolates #1–4 and #5) and omasum/fecal samples (isolates #21-22 and 17–20), their genetic associations were examined by pulsed-field gel electrophoresis (PFGE). This approach then revealed the identical macrogenotypes among four tongue isolates and one liver isolate that were slaughtered almost consecutively (Figure 1). Likewise, two omasum isolates (isolates #21-22) also exhibited identical PFGE pattern with fecal isolates #17–20 that were obtained at the same date (24/Aug/2006) (Figure 1). These suggested that cross-contamination at slaughter might be a possible factor for this dissemination.

Moreover, four fecal isolates #8-9 and #27-28 exhibited identical PFGE patterns with close phylogenic lineages (Figure 1). Because these isolates were originated from animals fed at the same farm (farm F, Table 1), it could be considered that this O157 might be widely disseminated at that farm continuously. Indeed, a previous study demonstrated that a part of bovine animals shed high doses of O157 longitudinally (so-called “super shedders”) [14]. A recently trialed vaccine against type III secreted proteins [15] might be effective for the reduction of such continuous spread of this pathogen at farms. In this relation, two beef carcass isolates #40 and 41 showed different PFGE genotypes although they were slaughtered almost consecutively (Figure 1). It is likely that the super shedders can have a disproportionate effect on the animal's hide and subsequent carcass contamination while low-shedding animals are also linked to the contamination on beef carcass [16]. Minimizing or eliminating the super shedding animals would thus contribute, at least in part, to the reduction of contamination with O157 STEC on beef carcasses.

As a molecular classification tool, we used PFGE to genetically discriminate representative STEC O157 isolates. The PFGE types are known to be altered during passage in bovine intestine [17], and therefore the identical PFGE patterns of O157 isolates from offal and feces of bovines at slaughter suggest that this dissemination of O157 might be due to animal-to-animal contact at preslaughter (including farm environment) and/or cross-contamination at slaughter, respectively. In this relation, Arthur et al. (2011) reported that the O157 cells could survive on cattle hides for up to 9 days after infection [18], suggesting that animal-to-animal contact at preslaughter might be also one of the important factors for the bacterial dissemination. In addition, the detection of STEC O157 at the surface of tongues and omasum further provided an idea that this pathogen could be disseminated at slaughter by diapedesis of oral and gastric contents to the surroundings. In this support, Bergholz and Whittam reported the superior ability of STEC O157 in acid resistance to the other serotypes of STEC [19].

## 4. Conclusion

Our data showed the prevalence of STEC O157 in bovine feces and offal at slaughter in Japan. The genetic characterization reposes the importance for the proper salinity control at bovine (pre)slaughtering processes to prevent spread of STEC O157 to beef carcasses and offal.

## Figures and Tables

**Table 1:** Summary of STEC O157 isolates obtained in this study.

Isolate number	Date of slaughter	Animal number^∗1^	Place^∗2^	Farm^∗2^	Source	Virulence gene		
						*stx *	*eaeA *	*hlyA *
1	6/Jul/2006	206	i	A	Tongue	2c	+	+
2	6/Jul/2006	207	ii	B	Tongue	2c	+	+
3	6/Jul/2006	208	ii	B	Tongue	2c	+	+
4	6/Jul/2006	210	iii	C	Tongue	2c	+	+
5	6/Jul/2006	207	ii	B	Liver	2c	+	+
6	20/Jul/2006	842	iv	D	Omasum	1 + 2c	+	+
7	27/Jul/2006	1202	v	E	Feces	2c	+	+
8	27/Jul/2006	1207	v	F	Feces	2c	+	+
9	27/Jul/2006	1208	v	F	Feces	2c	+	+
10	27/Jul/2006	1242	iii	G	Feces	2c	+	+
11	27/Jul/2006	1257	i	H	Feces	2c	+	+
12	27/Jul/2006	1263	i	I	Feces	1		
13	28/Jul/2006	1315	vi	J	Feces	2c	+	+
14	28/Jul/2006	1316	vi	J	Feces	2c	+	+
15	28/Jul/2006	1318	vi	J	Feces	2c	+	+
16	23/Aug/2006	834	vii	K	Feces	2c	+	+
17	24/Aug/2006	852	iii	L	Feces	2c	+	+
18	24/Aug/2006	868	vii	M	Feces	2c	+	+
19	24/Aug/2006	869	vii	M	Feces	2c	+	+
20	24/Aug/2006	883	vii	N	Feces	1 + 2c	+	+
21	24/Aug/2006	851	iii	O	Omasum	2c	+	+
22	24/Aug/2006	853	iii	L	Omasum	2c	+	+
23	25/Aug/2006	936	viii	P	Feces	2c	+	+
24	25/Aug/2006	937	viii	P	Feces	2c	+	+
25	30/Aug/2006	1048	v	E	Feces	2c	+	+
26	30/Aug/2006	1050	v	Q	Feces	2c	+	+
27	30/Aug/2006	1057	v	F	Feces	2c	+	+
28	30/Aug/2006	1058	v	F	Feces	2c	+	+
29	30/Aug/2006	1068	vii	R	Feces	2c	+	+
30	9/Sep/2006	383	i	I	Feces	1		
31	9/Sep/2006	388	i	S	Feces	2c	+	+
32	9/Sep/2006	389	i	S	Feces	2c	+	+
33	9/Sep/2006	392	i	T	Feces	2c	+	+
34	9/Sep/2006	399	vii	U	Feces	2c	+	+
35	9/Sep/2006	400	vii	U	Feces	2c	+	+
36	9/Sep/2006	401	vii	U	Feces	2c	+	+
37	9/Sep/2006	424	ix	V	Feces	2c	+	+
38	14/Sep/2006	601	viii	P	Feces	2c	+	+
39	30/Sep/2006	1152	ix	W	Feces	2c	+	+
40	17/Aug/2006	635	vii	N	Carcass	2c	+	+
41	24/Aug/2006	890	vii	N	Carcass	1 + 2	+	+

^∗1^Animals were numbered monthly according to the slaughtering order. ^∗2^The places (prefectures) and farms, where the animals were fed, were shown in Arabic numerals or alphabetic orders.
